# Surgical management of posterior fossa metastases

**DOI:** 10.1007/s11060-016-2254-2

**Published:** 2016-09-12

**Authors:** Geraint J. Sunderland, Michael D. Jenkinson, Rasheed Zakaria

**Affiliations:** 1 Department of Neurosurgery, The Walton Centre NHS Foundation Trust, Lower Lane, Fazakerley, Liverpool, L97LJ UK; 2 Institute of Translational Medicine, University of Liverpool, Liverpool, UK; 3 Institute of Integrative Biology, University of Liverpool, Liverpool, UK

**Keywords:** Brain metastasis, Cerebellar metastasis, Posterior fossa metastasis, Neurosurgery

## Abstract

The diagnosis of brain metastases is associated with a poor prognosis reflecting uncontrolled primary disease that has spread to the relative sanctuary of the central nervous system. 20 % of brain metastases occur in the posterior fossa and are associated with significant morbidity. The risk of acute hydrocephalus and potential for sudden death means these metastases are often dealt with as emergency cases. This approach means a full pre-operative assessment and staging of underlying disease may be neglected and a proportion of patients undergo comparatively high risk surgery with little or no survival benefit. This study aimed to assess outcomes in patients to identify factors that may assist in case selection. We report a retrospective case series of 92 consecutive patients operated for posterior fossa metastases between 2007 and 2012. Routine demographic data was collected plus data on performance status, primary cancer site, details of surgery, adjuvant treatment and survival. The only independent positive prognostic factors identified on multivariate analysis were good performance status (if Karnofsky performance score >70, hazard ratio (HR) for death 0.36, 95 % confidence interval (CI) 0.18–0.69), adjuvant whole brain radiotherapy (HR 0.37, 95 % CI 0.21–0.65) and adjuvant chemotherapy where there was extracranial disease and non-synchronous presentation (HR 0.51, 95 % CI 0.31–0.82). Patients presenting with posterior fossa metastases may not be investigated as thoroughly as those with supratentorial tumours. Staging and assessment is essential however, and in the meantime emergencies related to tumour mass effect should be managed with steroids and cerebrospinal fluid diversion as required.

## Introduction

Brain metastases (BM) occur in 15–30 % of all cancers [1]. They are associated with significant morbidity and have a poor prognosis if untreated [2]. Historically the diagnosis of brain metastases was considered a pre-terminal event, representing the end-stage of uncontrollable primary disease and treatment was limited to palliative whole brain radiotherapy (WBRT) only [3]. The incidence of BM has increased in recent years as a consequence of improved imaging, but also an effect of improved survival from primary tumours [4]. Advances in the management of extracranial disease have led to a more aggressive approach in the management of brain metastatic disease.

The most common primary site of malignancy for brain metastasis is lung (20–40 %) followed by breast (5–17 %) and melanoma (7–11 %) with renal, colorectal and gynaecological cancers making up the majority of the remaining [5–7].

There is no curative treatment, even for those patients with an apparently isolated metastasis, therefore the aim of treatment is extended, quality survival. Previously nihilistic management has been superseded by aggressive approaches to the control of metastatic disease involving both surgical resection and stereotactic radiosurgery (SRS). These are associated with appreciable improvement in both progression-free and overall survival [8–11]. Surgical resection of a single large and symptomatic lesion in the presence of additional cranial disease has also been shown to confer a survival advantage and symptomatic improvement when compared with whole brain radiotherapy (WBRT) alone [11, 12]. Stereotactic radiosurgery (SRS), likewise has been shown to confer survival advantage and the addition of WBRT improves local disease control, although it does not appear to increase overall survival [13–15].

Improved imaging modalities, particularly the routine use of contrast-enhanced MRI, have allowed us to gain a more accurate impression of the disease burden and this has resulted in a decreased incidence of solitary metastatic disease, with 50–75 % of patients now presenting with multiple lesions [16, 17].

**Fig. 1:**
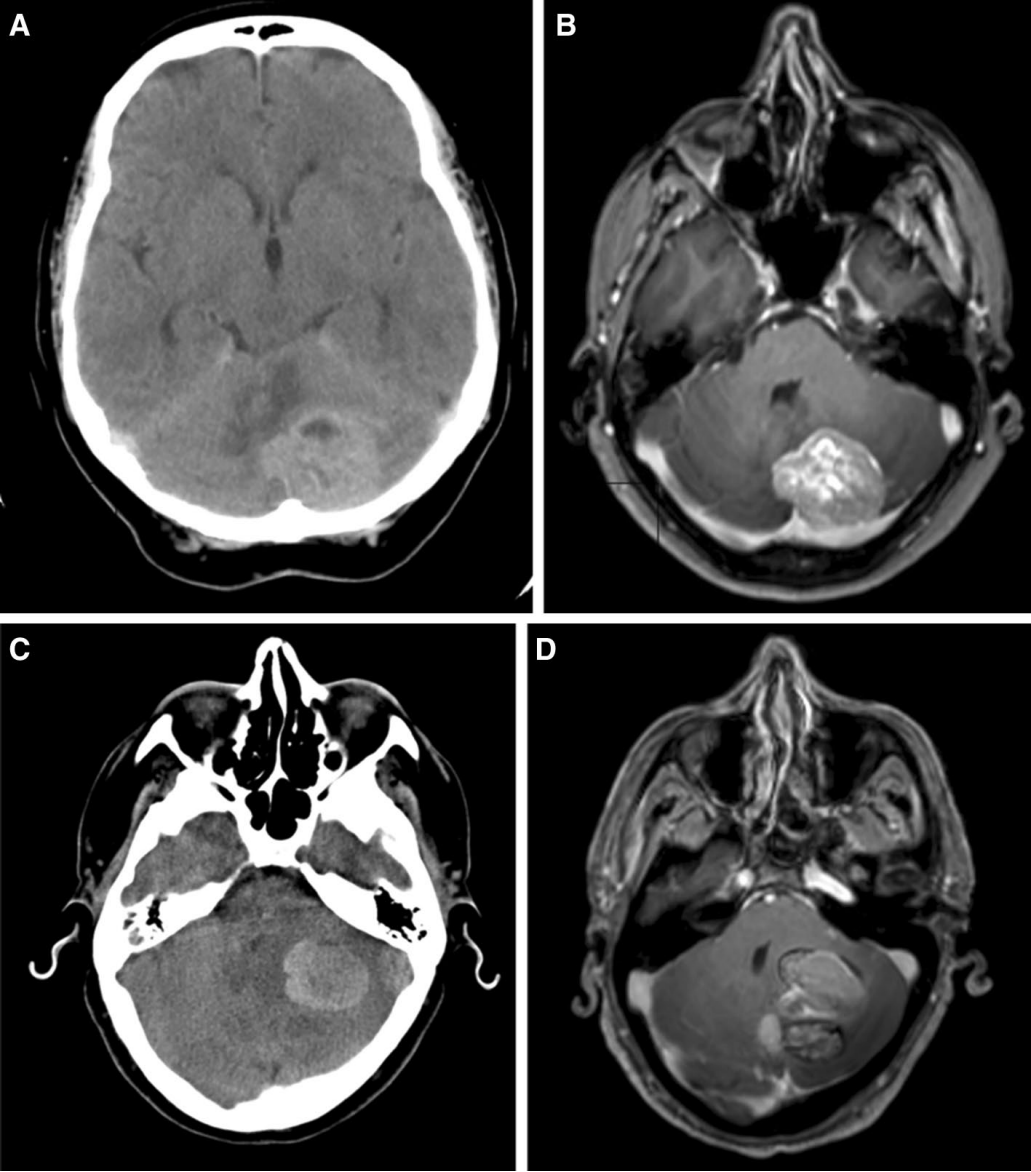
Illustrative cases. Case 1 images (**a**) and (**b**) 54 year old female. PMH Breast cancer (Her2 positive) Treated with mastectomy 1 year ago plus adjuvant chemotherapy (trastuzumab). Presented with 2 weeks of headaches and unsteadiness. CT brain revealed (**a**) large solid left cerebellar tumour, confirmed on MRI (**b**). KPS 90 pre-operatively. CT staging pre-op showed local lymph node involvement but no other metastatic disease. Underwent craniotomy and gross total resection with adjuvant WBRT and further systemic chemotherapy. Re-presented with similar symptoms 21 months later and MRI showed recurrent tumour at the same site. CT staging showed no evidence of extracranial disease. Further craniotomy and gross total resection performed. No further adjuvant therapy given. Gradual deterioration 10 months after second surgery and died 36 months after initial diagnosis. Case 2 images (**c**) and (**d**) 67 year old male. PMH Testicular cancer - orchidectomy 12 years ago. Presented with increasing confusion, headache, unsteadiness and falls. Acute deterioration the previous day. KPS 50 on arrival. CT head revealed 36 × 25 mm left cerebellar haemorrhagic tumour with triventricular hydrocephalus (**c**). Transferred to regional neurosurgical unit and external ventricular drain (EVD) inserted. MRI confirmed solitary bulky left cerebellar (**d**). Gross total resection performed later that admission, histology revealed metastatic carcinoma. Post- operatively the patient developed bulbar dysfunction and swallowing difficulty. Staging CT performed post-operatively revealed metastatic lung cancer with mediastinal and liver metastases. EVD reinserted due to post-operative hydrocephalus. 5 days of palliative WBRT administered as an inpatient. Continued deterioration and the patient died in hospital 5 weeks after surgery

The posterior fossa is an important site for BM with 20 % of lesions observed to occur here in historical series [18]. BMs in this region exerting mass effect present with a characteristic triad of symptoms consisting of headache, ataxia and nausea/vomiting. Neurosurgeons and oncologists are wary of posterior fossa BMs in particular due to the risk of acute obstructive hydrocephalus with rapid coma and death if this not managed expediently [19]. As a result, there has been a trend to manage these cases as emergencies by resecting the tumour. This may be preceded or associated with CSF diversion, particularly in those patients who present with a rapid deterioration out of hours [20, 21]. This expedited approach to surgery often comes at the expense of a full pre-operative work up including a staging CT scan (chest, abdomen and pelvis) and information about likely prognosis and options for systemic treatment. See illustrative cases in Fig. 1. The aim of this study was to assess the outcome of surgery in patients with posterior fossa brain metastases in a single centre in order to identify factors that may assist in case selection in future.

## Materials and methods

Patients who underwent posterior fossa surgery for metastatic tumours between 2007 and 2012 were identified from a computerised operative database (MD analyse v3.11). The institution is a regional neurosciences centre serving a population of approximately 3.5 million. Demographic and clinical information was collected from operative notes, pathology reports, clinical case notes, correspondence, radiological images and HES mortality data. Only adult patients (16 years+) were included for study. Subjects were stratified by good performance status defined by convention as KPS > 70, the ability to self-care. The primary endpoint was overall survival, which was defined as the time from diagnosis of brain metastasis to death. Secondary endpoints were fitness to receive adjuvant WBRT or systemic chemotherapy and surgical complications (infection, return to theatre, post-operative hydrocephalus requiring CSF diversion within 28 days of resection). Data analysis was performed using SPSS (Version 22.0. Armonk, NY: IBM Corp). Univariate time-to-event analyses were performed using the log rank test and multivariate analysis by Cox’s proportional hazards model.

## Results

In total 92 patients were included, 60 female and 32 male, with a median age of 59 years (range 37–76). The most common primary tumours were lung (33), breast (21), colorectal (8) and renal (7). 33 patients (36 %) presented synchronously with a solid organ cancer and BM. Of the 59 patients (64 %) with previously diagnosed primary malignancy (metachronous presentation) 40 (69 %) had stable disease and 19 (31 %) had progressive disease (Table 1).

**Table 1:** Data for 92 patients surgically treated for posterior fossa metastases

Patients n = 92							
Median age (range)	60 (37–76)						

Variable	Classification	Number	%				
Primary site	Lung	38	41.2				
	Breast	21	22.8				
	Colorectal	8	8.7				
	Renal	7	7.6				
	Melanoma	4	4.3				
	Other	14	15.4				

	Classification	Number	%	Median OS—months (95 %CI)	Log rank (P value)	Cox regression P value	Hazard ratio—death (95 % CI)
Age							
<65		67	72.8	7.00 (5.42–8.58)	3.781 (0.052)		
>65		25	27.2	4.00 (2.12–5.88)			
Gender							
M		32	34.8	5.00 (4.12–7.88)	3.276 (0.07)		
F		60	65.2	6.00 (2.84–7.15)			
Adjuvant radiotherapy							
Y		63	73.3	8.00 (5.9-10.09)	17.525 (<0.001)*	0.001*	0.374 (0.213–0.654)
N		23	26.7	2.00 (1.12–2.88)			
Adjuvant chemotherapy							
Y		41	48.2	9.00 (6.31–11.69)	8.951 (0.003)*	0.006*	0.507 (0.314-0820)
N		44	51.8	3.00 (2.31–3.69)			
Number of cranial metastases							
Single		73	79.3	6.00 (4.36–7.64)	0.371 (0.54)		
Multiple		19	20.7	5.00 (1.59–8.41)			
Karnofsky performance status							
>70		68	74.7	7.00 (4.08–9.02)	21.042 (<0.001)*	0.002*	0.356 (0.184–0.689)
<70		23	25.3	2.00 (0.74–3.26)			
Synchronous presentation							
Y		33	36.3	6.00 (4.80–7.20)	5.297 (0.021)*	0.164	1.423 (0.866–2.338)
N		58	63.7	7.00 (0.89–13.11)			
Extracranial metastases							
Y		50	54.9	6.00 (2.91–9.09)	0.096 (0.756)		
N		41	45.1	6.00 (4.87–7.13)			
Primary disease status							
Stable		40	69	7.00 (0.89–13.11)	0.001 (0.973)		
Progressive		18	31	6.00 (2.15–11.85)			
RPA class							
I		22	23.9	5.00 (3.10-10.91)	2.656 (0.265)		
II		64	69.6	6.00 (4.79–7.63)			
III		5	5.4	4.00 (0.53–7.42)			

*Asterisk* denotes statistical significance p < 0.05

**Fig. 2:**
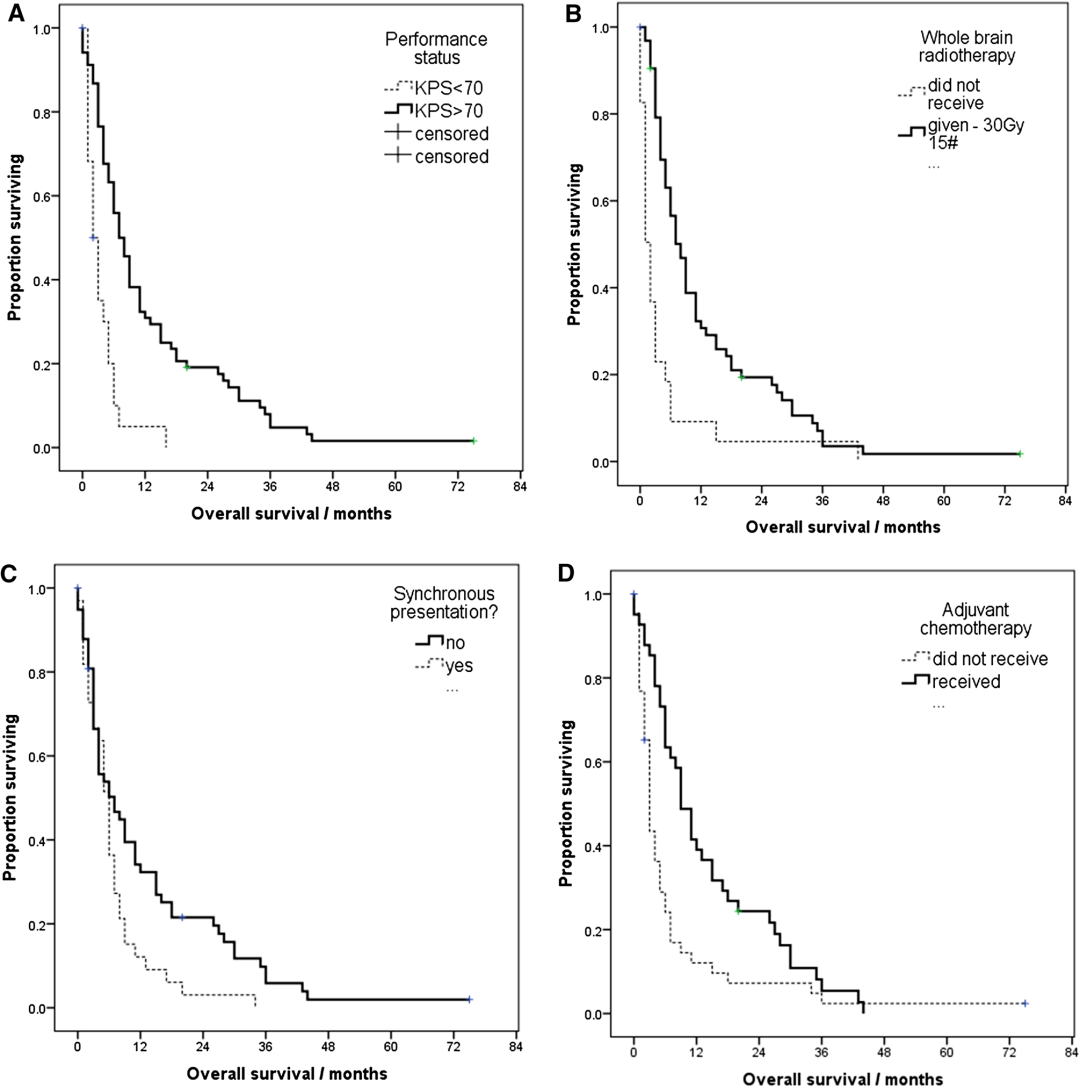
**a** Kaplan–Meier survival analysis for patients with good Karnofsky performance status (>70) versus those with poor (<70) (log rank = 21.042, p < 0.001). **b** Kaplan–Meier survival analysis for patients receiving adjuvant WBRT versus those who did not receive WBRT. (log rank test = 17.525, p < 0.001). **c** Kaplan–Meier survival analysis for synchronous versus metachronous presentation. (log rank test = 5.97, p = 0.021). **d** Kaplan–Meier survival analysis comparing patients who received adjuvant chemotherapy for synchronous or uncontrolled systemic disease versus patients who did not receive this treatment. (log rank test = 8.951, p = 0.003)

Overall, 74 patients (80 %) underwent gross total resection (GTR), 13 (14 %) subtotal resection (STR) and 5 (6 %) underwent biopsy only. The median interval from diagnosis to surgery was 8 days (range: 0–90). Seven patients (7.6 %) required pre-operative CSF diversion for acute hydrocephalus. Median overall survival (OS) was 6.00 months (95 % CI 4.37–7.63). Median Karnofsky Performance Status (KPS) as assessed by the clinician at presentation was 80 and 75 % of patients had a KPS > 70 [22]. Good performance status was associated with longer survival; OS was 7.00 months (95 % CI 4.98–9.02) for those with KPS > 70, versus 2.00 months (95 % CI 0.74–3.26) for those with KPS < 70 (log rank = 21.042, p < 0.001) (Fig. 2a).

Overall, 27 % of operated patients did not go on to receive WBRT due to poor performance status and operative complications. The median time interval from neurosurgery to WBRT was 22 days (range 8–64). Receiving adjuvant WBRT increased OS significantly; 8.00 months (95 % CI 5.90–10.09) if given, versus 2.00 months (95 % CI 1.12–2.88) if not, log rank test = 17.525, p < 0.001 (Fig. 2b).

Data on adjuvant chemotherapy was available for 85 of 92 patients of which 41 (48.2 %) went on to receive adjuvant chemotherapy for their primary cancer. These patients had a 6-month improvement in their median OS compared with patients who did not go on to receive chemotherapy from 3.00 months (95 % CI 2.31–3.69) to 9.00 months (95 % CI 6.31–11.69) (log rank test = 8.951, p = 0.003) (Fig. 2d).

Median OS for patients presenting with synchronous brain metastases was 6 months (95 % CI 4.8–7.2) compared to 7 months (95 % CI 2.1–11.8) for those with metachronous presentation, (log rank = 5.297, p = 0.021, Fig. 2c). 45 patients presented with either synchronous primary and metastatic disease, or with metachronous presentation with progressive primary disease. Data was available for 39 of these patients and showed that 22 (56 %) were considered unfit for systemic chemotherapy due to poor performance status. Six of these patients (15.4 %) were too unfit for WBRT when assessed by oncologists after surgery.

Variables that did not have a statistically significant bearing on prognosis included sex, age > 65 years, multiple cranial lesions, extracranial disease and control of primary cancer. RPA class similarly did not appear to have a significant influence on median OS (Table 1).

Multivariate analysis using Cox proportional hazards model suggested that good performance status (KPS > 70), progression to adjuvant WBRT and chemotherapy were independently associated with a good outcome, HR for death 0.36 (95 % CI 0.18–0.69), 0.37 (95 % CI 0.21–0.65) and 0.51 (95 % CI 0.31–0.82) respectively. Synchronous presentation was not independently associated with overall survival based on multivariate analysis, HR 0.16 (95 % CI 0.86–2.34) (Table 1).

The 28 day mortality was 7.6 % (n = 7) with a perioperative morbidity of 22.8 % (n = 21). These included 6 deep wound infections; 5 abscesses requiring re-operation and 1 episode of ventriculitis. There were 14 cases of post-operative hydrocephalus, half of which required permanent CSF diversion (5 VP shunt, 1 Ommaya reservoir and 3 endoscopic third ventriculostomy).

Fewer patients with post-operative complications (62 %)went on to receive WBRT compared to those without complications (70 %). There was no significant delay in the time from surgery to receiving WBRT between these groups (Student *t* test, p = 0.206).

Furthermore, comparison of survival of posterior fossa BM patients with a series of supratentorial BM patients from the same unit and time period revealed poorer prognosis for patients with infratentorial disease even when matched for KPS and progression to adjuvant therapy (WBRT and chemotherapy). On univariate analysis posterior fossa location showed a non-significant trend towards negative prognosis, median OS 6.1 months (95 % CI 4.5–7.8) versus 9.3 months (95 % CI 6.0–12.6) for patients with supratentorial metastases (log rank = 1.219 p = 0.270). Multivariate analysis including performance status (KPS > 70) and progression to adjuvant therapy using Cox proportional hazards model however revealed a significant negative prognostic effect of posterior fossa location, HR 1.43 (95 % CI 1.04–1.98) p = 0.029.

## Discussion

Our single centre series of 92 cases has shown that patients with posterior fossa BM have a poor prognosis, with median OS of 6 months. Factors associated with a longer survival were KPS > 70 at presentation and progression to adjuvant WBRT and systemic chemotherapy for uncontrolled extracranial disease.

Despite the growing arsenal of treatments for systemic cancer, the management of brain metastases has remained largely unchanged for over 20 years. Chemotherapy for BM is of limited utility due to the poor penetrance of pharmacological agents into the central nervous system. Treatment therefore consists for the most part of surgical resection of the visible lesion or lesions in oligometastatic cases followed by fractionated WBRT or SRS.

SRS presents an appealing treatment option for posterior fossa metastases but concerns regarding its safety exist. Following treatment, tumours and peritumoural tissue can swell and exert mass effect on surrounding structures [23–25]. This precludes the use of SRS in those patients with a crowded posterior fossa and established or impending hydrocephalus.

A previous case series published in 2003 found survival with posterior fossa metastases to be comparable to that of patients with supratentorial metastases when the groups were stratified by RPA class [26]. This runs counter to a number of other case series which found worse prognosis associated with infratentorial location [20, 27–29]. Our series demonstrates a median OS of 6 months for all operated posterior fossa BMs. Grouping patients by RPA class, proven to be an accurate prognostic indicator when considering supratentorial disease, showed no correlation with survival in our series. Furthermore, comparison of survival of posterior fossa BM patients with a series of supratentorial BM patients from the same unit and time period revealed poorer prognosis for patients with infratentorial disease even when matched for KPS and progression to adjuvant therapy (WBRT and chemotherapy).

Initial series of cerebellar tumour resections performed in the late nineteenth century had mortality rates of up to 70 %. These improved from the 1920s to 20 % with improvements in surgical technique, anatomical understanding and perioperative care [19]. The development of microsurgical techniques in the last 50 years has resulted in a further improvement in operative mortality. Postoperative CSF leak is of particular concern due to the risk of deep infection and meningoencephalitis and ventriculitis. Most commonly, this is a result of altered CSF flow dynamics and post-operative hydrocephalus. Patients frequently require further CSF diversion procedures to manage this, extending hospital stay and further increasing the surgical stress on the patient. Shunt procedures are themselves associated with infection risk and risk of revision within 30 days may be as high as 13 % in some series [30]. In addition, shunt systems may block, particularly in the presence of blood and high CSF protein associated with metastatic disease.

Endoscopic third ventriculostomy (ETV) presents an attractive treatment option to treat obstructive hydrocephalus avoiding complications associating with VP shunts. The literature for ETV in metastatic disease is limited, its use is mainly established for paediatric posterior fossa tumours and benign aqueduct stenosis in adults [31, 32]. Success rates of shunt independence are quoted between 50 and 90 % for all indications, with most series reporting success rates of around 70 % [33–38]. In our series 3 ETVs were performed, 2 of which failed requiring subsequent VP shunt. High CSF protein and haemorrhage from metastatic tumours are proposed as the mechanism predisposing occlusion of ventriculostomy in this population.

Interpretation of our data is limited by the study’s retrospective design, nevertheless this represents an unselected series of patients operated for posterior fossa metastases. The improved survival associated with progression to adjuvant therapy may reflect a surrogate effect due to these patients having a better baseline performance status. Future prospective studies would be of use to guide treatment, specifically to further delineate those factors that accurately predict improved survival. Likewise, further information on why patients with infratentorial tumours do worse than their supratentorial counterparts would assist in the selection of patients who are likely to benefit from surgical intervention. In the acute setting, even limited information about systemic disease status would help in selection those patients that would benefit from direct surgical resection of a posterior fossa metastasis, for example, known breast cancer with good performance status. This contrasts with the patient with widespread metastatic disease and poor performance status in whom CSF diversion may be more appropriate to determine whether they will benefit from surgical resection and adjuvant therapy.

## Conclusions

We present a retrospective single-unit case series of 92 patients undergoing surgery for posterior fossa metastases further confirming the dismal prognosis associated with this diagnosis. Good performance status (KPS > 70) and progression to receive adjuvant WBRT and/or chemotherapy were the only independently significant prognostic factors. In view of the high morbidity and mortality associated with surgical resection, we propose that these patients should be managed with corticosteroids and temporary CSF diversion if required in the first instance rather than proceeding to emergency surgical resection wherever possible. This allows sufficient time to complete a full pre-operative work-up of all patients prior to surgery including cancer staging. In doing this it should be possible to identify those patients who will benefit from surgery and spare those who will not the associated operative morbidity.
